# STEAM in education: a bibliometric analysis of performance and co-words in Web of Science

**DOI:** 10.1186/s40594-021-00296-x

**Published:** 2021-06-25

**Authors:** José-Antonio Marín-Marín, Antonio-José Moreno-Guerrero, Pablo Dúo-Terrón, Jesús López-Belmonte

**Affiliations:** 1 Department of Didactics and School Organization, University of Granada, 18071 Granada, Spain; 2 Department of Didactics and School Organization, University of Granada, 51001 Ceuta, Spain; 3 CEIP Principe Felipe, Ministry of Education and Vocational Training, Ceuta, 51003 Ceuta, Spain

**Keywords:** STEAM, SciMAT, Education, Bibliometrics

## Abstract

**Background:**

Emerging methodologies that apply and integrate science, technology, engineering, art, and math (STEAM) in education have appeared in recent years as a pedagogical alternative providing more holistic and attractive education.

**Method:**

The research methodology used in this work is of a bibliometric nature. Specifically, an academic performance analysis and a co-word analysis has been carried out. The term STEAM was analyzed in the Web of Science (WoS) database. The WoS programs Analyze Results, Creation Citation Report, and SciMAT were used. A total of 1116 manuscripts were analyzed.

**Results:**

The results show that studies in the field education of STEAM began in 2006 and have continued uninterruptedly up to the present day, although interest generated in the scientific community has been irregular.

**Conclusions:**

It can be concluded that STEAM studies have not had an established and robust line of research over time, although it can be observed that the trends in this aspect are focused on the scientific branch of education. In addition, the topics of study on STEAM include points related to gender differences, the influence of STEAM on people of different races, the skills developed by students, and training teachers to implement teaching and learning processes with STEAM.

## Introduction

Digital media and tools are increasingly being introduced into society. The increasing of technological projects in these recent years is notable, although their theoretical foundations are limited to the field of science (Chu et al. 2018). For this reason, studies and practices focused on other disciplines related to science, technology, engineering, art, and math (STEAM) have been conducted (Angel and Salgado 2018; Bush et al. 2020; Colucci-Gray et al. 2019; Dolgopolovas and Dagiene 2021; Lin and Tsai 2021; Mengmeng et al. 2019; Perignat and Katz-Buonincontro 2018; Togou et al. 2019; Webb and LoFaro 2020) and share a relationship with the science, technology, engineering, and mathematics (STEM) approach (Chu et al. 2018; Greca et al. 2021; Herro et al. 2017) with a key element integrated, the acronym “A,” alluding to the arts and creativity (Conradty and Bogner 2020; Herro et al. 2017), allowing one to know and experience the world, enabled by art forms, practices, or even pedagogies (Colucci-Gray et al. 2019). In addition, art develops mathematical and scientific content and procedures in order to encourage, among others, mathematical competence (Angel and Salgado 2018) by providing a suitable method to successfully retain knowledge (Bush et al. 2020; Colucci-Gray et al. 2019; Dolgopolovas and Dagiene 2021; Lin and Tsai 2021; Mengmeng et al. 2019; Perignat and Katz-Buonincontro 2018; Salmi et al. 2020; Togou et al. 2019; Webb and LoFaro 2020). Moreover, it shares a relationship with the STEM approach “Science, Technology, Engineering, Mathematics” (Chu et al. 2018; Greca et al. 2021; Herro et al. 2017) where a key element is integrated, the acronym “A,” alluding to the arts and creativity (Conradty and Bogner 2020; Herro et al. 2017), allowing to understand and experience the world, enabled by art forms, practices, or even pedagogies (Colucci-Gray et al. 2019).

In the digital society and economy that we are immersed in, there is an increasing demand for professions linked to the use of technological media and tools (Anisimova et al. 2020), as machines are replacing human labor (Anito and Morales 2019), with programmers, systems engineers, biotechnologists, laboratory technicians, and project leaders being among the most prominent examples (Anisimova et al. 2020). The lack of competent workers choosing STEM careers has promoted a STEAM movement as an alternative to solve problems while taking advantage of creative and collaborative skills in learning spaces to increase interest and participation (Herro et al. 2017) in mathematical, scientific, and artistic fields (Angel and Salgado 2018). For this reason, the educational field is the future line, which prepares young people in a productive and global way, facing a social, economic, political, environmental, and cultural heritage crisis. Emerging skills are needed in the labor market to ensure the success of students in their professional life (Anito and Morales 2019; Max et al. 2020; Taylor 2018). Art develops skills, content, and procedures in order to promote, among others, mathematical competence (Angel and Salgado 2018) or social and civic competence (Burnard and Colucci-Gray 2019), providing an adequate method to successfully retain knowledge (Salmi et al. 2020). Teaching and learning must be understood as complementary elements, not separately, so that it can be “taught to learn,” from the learner’s point of view, in an epistemological way (Biesta 2015).

The arts, as a complement to STEM, have a very broad meaning, from general forms such as painting, drawing, and photography, among others, to more particular ones, such as the performing arts, makerspaces, aesthetics, or crafts (Colucci-Gray et al. 2017). These disciplines allow human beings to relate to each other, creating dialogue, discussion, reasoning, thoughts, and ideas, which allow for experimenting with a constructivist methodology. Through the arts and sciences, students are able to imagine and reflect on what the collective future society will be like, and to change another form of learning in schools, which translates the theory of thought into practice (Burnard and Colucci-Gray 2019). There are increasing social and environmental discourses based on science and technology that affect the nature and ecology of the planet, which extends to the material, affective, and cognitive relationships of human beings. The interest in the sustainability of the planet allows to study the human being in general and its characteristics, adopting a post-humanist thought in which STEAM, through creativity, science, and art, makes connections between mind and nature that are, therefore, integral ways in which, as individuals, we face the world (Colucci-Gray 2019). This way of including art allows an ontological study. In this sense, Ruiz et al. (2020) highlighted the motivation and involvement that STEAM creates in students by designing and creating sustainable cities and robots for our future.

These social and technological needs of the 21st century call for a redefinition of teaching and learning models (Casado and Checa 2020). Historically, the education system has required students to choose between the arts and sciences (Jesionkowska et al. 2020). Bringing together STEM subjects and the arts in active learning provides a more holistic and engaging education (Herro et al. 2017; Jesionkowska et al. 2020). Through multisensory technologies and makerspaces, student engagement and learning outcomes are promoted (De la Garza 2019; Kajamaa and Kumpulainen 2020; Taljaard 2016), thus calling for an educational reform with this improved version of STEM, resulting in a new teaching model (Bazler and Van Sickle 2017; Wu et al. 2021) that is transdisciplinary, interdisciplinary and multidisciplinary, along with artistic integration (Harris and Bruin 2017; Kim 2016; Perignat and Katz-Buonincontro 2018), with a planning adapted to content, strategies, pedagogical practices and evaluation (Cook et al. 2020). The implementation of STEAM projects in the educational field (STEAM-EDU) has emerged in the last decade (Lin and Tsai 2021) and is gaining a lot of attention (Bush et al. 2020) in current curricula, which broadens their traditional objectives and increasingly promotes creativity (Conradty et al. 2020). Furthermore, it has a positive effect on student’s motivation and increases self-efficacy (Conradty and Bogner 2020) in makerspaces, where students imagine, explore, experiment, test, manipulate, discuss, and speculate (Conradty et al. 2020). Learning through STEAM-EDU has been recognized as a key driver of progress (Mengmeng et al. 2019) and can change the direction of future learning in the context of this new interactive era (Tan et al. 2020).

The technological and digital revolution has created new educational opportunities (López-Belmonte et al. 2021), including multisensory and immersive technologies (Jesionkowska et al. 2020). Augmented reality, virtual reality, artificial intelligence (AI), robotics, simulations, virtual field trips, and 3D printing (Casado and Checa 2020; Chu et al. 2018; How and Hung 2019; Togou et al., 2019; Moreno-Guerrero et al. 2021a, 2021b, Rodríguez et al. 2020; Togou et al. 2019) are transforming education (Chien and Chu 2017) and have an emerging role in STEAM-EDU (Taljaard 2016). These disciplines contribute, in complementary ways, to learn (Segarra et al. 2018) and the student performance (Chen and Huang 2020). Computational thinking (CT) is an essential part of STEAM-EDU (Juskeviciene et al. 2020) because of the wide variety of areas and subjects it covers (Lytle et al. 2019). CT enables an understanding of how machines work and is a popular recent research topic among researchers (Juskeviciene 2020), where STEAM-EDU enhances students’ CT skills (Bati et al. 2018).

In this sense, AI and robotics stand out as novel fields of projection in STEAM-EDU (López-Belmonte et al. 2021). This is transferrable to the growing demand for jobs that can transform the economy and the labor market, according to the demands of an increasingly technological society. This implies new demands and requirements in the educational system in general (Marín-Marín et al. 2020), and in learning spaces in particular (Moreno-Guerrero et al. 2021a, 2021b; Tuomi 2018). These fields are useful as educational bridges for learning human-assisted CT, AI, and robotics skills in society (How and Hung 2019) and have great impact on the training of future teachers of physics, mathematics, technology, and visual arts, among others (Anisimova et al. 2020). It also ensures that students understand their implications and maximizes the potential opportunities to educate conscious and critical citizens in the future (Rodríguez et al. 2020). All of this marks a new era in the application of innovative and motivating teaching and learning processes (Casado and Checa 2020; Hinojo-Lucena et al. 2020) and, at the same time, improves attention (Campos et al. 2019), bringing these a priori distant disciplines closer to the curriculum (Suárez et al. 2018).

STEAM-EDU experiences develop critical and creative thinking, problem-solving skills, collaboration and discussion skills, roles and responsibilities, information skills, and literacy (Hadinugrahaningsih et al. 2017). This allows equity between both men and women, decreasing the gender gap and disparity in the field of science (Tan et al. 2020). Recently, women have been making their way into international engineering (Ng and Fergusson 2020; Oliveros-Ruiz 2019), and there is a significant increase in girls’ confidence and motivation in education (Diego-Mantecón et al. 2020).

Curricula emphasize competency-based learning from a constructivist paradigm, whereby students transform their knowledge (Casado and Checa 2020). Currently, there are not many substantial methodological changes in the classroom (Diego-Mantecón et al. 2020), and there is little research on STEAM methodologies and teaching resources among teachers (Greca et al. 2021; Herro et al. 2017; Hong et al. 2020), who need support, time, and experience to translate this methodology (Juskeviciene et al. 2020) into integrated lessons (Webb and LoFaro 2020). Makerspaces need to be experimental and encourage a great deal of reflection among teachers (Bassachs et al. 2020). This is the way to turn the teaching–learning process into a fully transdisciplinary curriculum (Wu et al. 2021) in the framework of an experiential curriculum (Knochel 2019) and should become a trend in the development of education at all educational stages (Lin and Tsai 2021). Student’s motivation and involvement in the educational process must be experiential and meaningful (Jesionkowska et al. 2020).

If the goal of education is to form citizens to be prepared for the future, using a creative and collaborative approach, arising from the reflection of the students themselves (Burnard and Colucci-Gray 2019), the application of these tools in the classroom must become a reality (Casado and Checa 2020). Teachers should strive to use STEAM-EDU approaches (Segarra et al. 2018) that produce meaningful use and understanding of the encompassed disciplines (Bassachs et al. 2020). This encourages teachers and students to cooperatively form an outcome and overcome the essential limits of a student-centered project (Kim 2016). Research on interdisciplinary curricula in STEAM-EDU (Lu and Ma 2019) can offer an educational path based on teaching methodologies that successfully demonstrate that art and creativity promote motivation and improve disciplines adapted to society and aimed at a sustainable development (Conradty et al. 2020).

### Justification and objectives

The research that has been carried out in this paper focuses on the analysis of STEAM-EDU. This is based on a bibliometric approach to the existing literature in the Web of Science (WoS) database (Carmona-Serrano et al. 2021). This database was chosen because it encompasses various areas related to the field of education, as well as being considered a prestigious database that hosts publications from the Journal Citation Reports (JCR) (Zhu and Liu 2020). Therefore, WoS is postulated as a relevant database to extract documents related to the state of the art analyzed in this study.

This research has an original and exploratory component. This is due to the fact that no work with the same characteristics as the one presented here can be found in the literature. That is, a study focused on an analysis of STEAM-EDU documents in WoS, in which both their performance and a scientific mapping (López-Belmonte et al. 2020a, 2020b) of the works linked to this art form are analyzed. Therefore, this research will generate new streams of knowledge as well as expand the literature on the subject. This will contribute to reducing the gap found in the impact literature. Therefore, this work will help researchers’ interest in this construct, as they will be able to visualize the full significance of STEAM-EDU and the lines of research it follows as well as the future trends that this concept will encompass (Moreno-Guerrero et al. 2020a, 2020b).

This work was based on the procedural guidelines at the analytical level of previous research (Soler-Costa et al. 2021), with the aim of using a study model that is accepted, contrasted, and verified by the scientific community. Based on the above, this study pursued the following objectives:

- To establish the significance of STEAM-EDU in scientific papers in WoS.
- To present the progress of STEAM-EDU in WoS scientific papers.
- To determine the most relevant focuses on STEAM-EDU in WoS scientific papers.
- To discover the most significant authors of research on STEAM-EDU in WoS scientific papers.

## Materials and methods

### Research design

The research methodology used in this work was bibliometric in nature (Moreno-Guerrero et al. 2020a, 2020b). This approach was chosen due to the potential it offers to accurately quantify and analyze the publications indexed in a database under study (Carmona-Serrano et al. 2020a, 2020b). In this sense, the development research design makes it possible to search, register, analyze, and predict the documents that revolve around a theme (Carmona-Serrano et al. 2020a, 2020b).

This study was also complemented with a co-word analysis (Herrera-Viedma et al. 2020). This type of analysis focuses on analyzing the keywords contained in the volume of documents reported in WoS. Specifically, this analysis makes it possible to make links between the topics investigated in the different publications on the construct under analysis. In addition, co-word analysis makes it possible to predict the topics that, in the near future, may be positioned as potentially relevant. This analysis is also used to draw up maps with nodes that determine the performance, the positions of terminological subdomains, and the development of a topic. Similarly, in this study, other indicators such as the h, g, hg, and q2 indices were taken into account for the analyses (López-Robles et al. 2019).

### Procedure

In this type of study, to avoid bias, a thorough procedure must be followed. For this, we took into account the considerations established in other studies (Moral-Muñoz et al. 2020), with the intention of carrying out all the required actions in an optimal manner. Specifically, the following processes have been carried out in this research (Montero-Díaz et al. 2018): choosing the study database = WoS; establishing the concept to be analyzed = STEAM; elaborating the search equation with all terms associated with the main construct = (“scienc*” AND “technolog*” AND “engine*” AND “art*” AND “math*”) OR (“STEAM”). This equation was carried out by selecting the search option focused on the THEME; this search strategy yielded a total of 98,183 publications. To refine this volume of documents, the search was narrowed down to the WoS categories focused on the educational field (Education Educational Research, Education Scientific Disciplines, Psychology Educational and Education Special), leaving a total of 1220 publications. In addition, various indexes were used (SCI-EXPANDED, SSCI, A&HCI, CPCI-S, CPCI-SSH, BKCI-S, BKCI-SSH, ESCI, CCR-EXPANDED, and IC). Similarly, several exclusion criteria were defined (López-Belmonte et al. 2020a, 2020b) in order to remove repeated documents (*n* = 37), poorly indexed documents (*n* = 59), publications released prior to 2006 (the year in which the term STEAM was coined), and documents referring to the year 2021 (*n* = 8), as the year had not ended. This reduced the document count to 1116 publications.

**Fig. 1:**
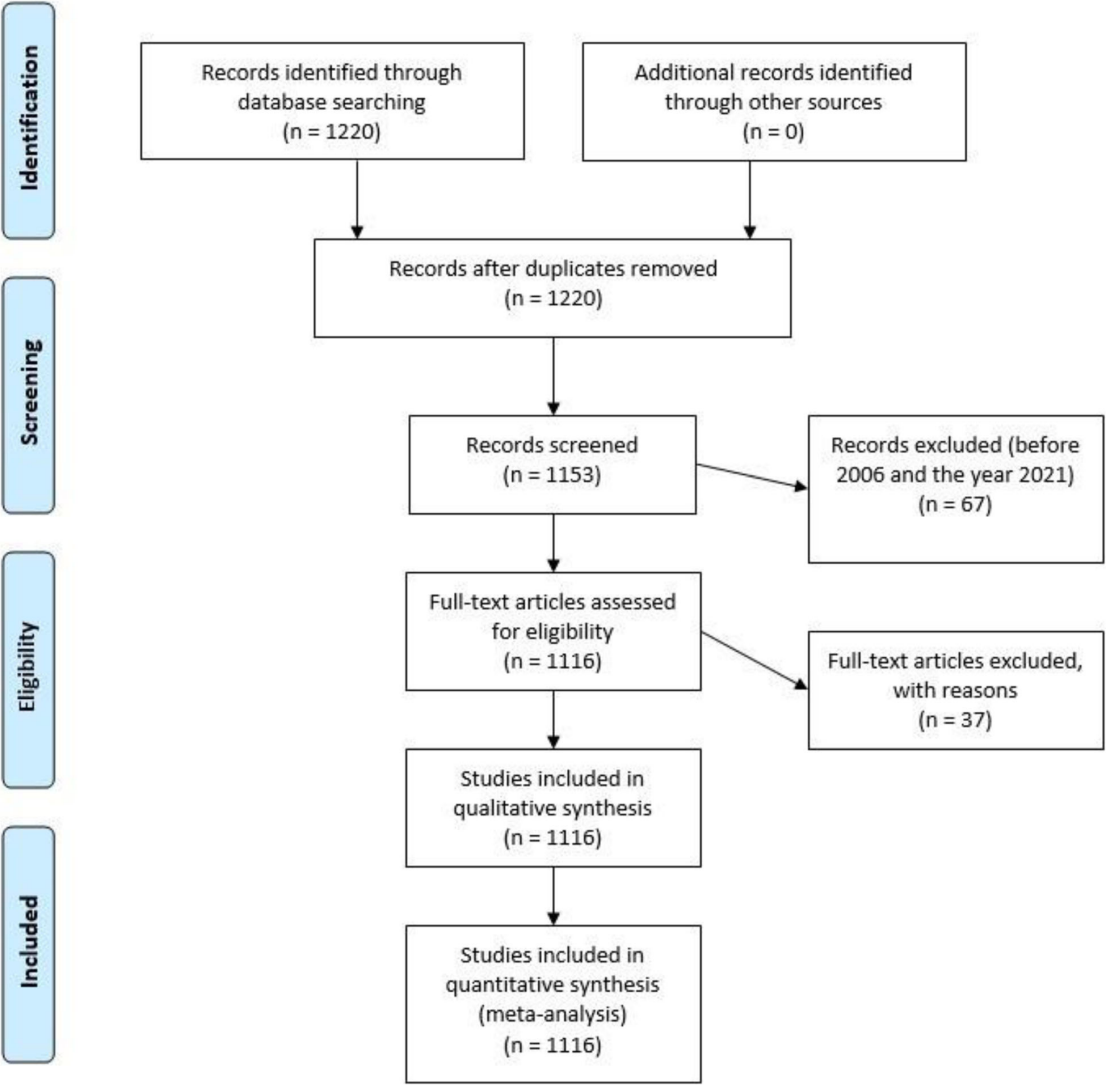
Flowchart according to the PRISMA declaration

In addition, for the review of publications, the standardized protocol of the PRISMA declaration was used, whose actions are shown in a flow chart (Fig. 1).

### Data analysis

Several programs were used to carry out the documentary analysis. Analyze Results and Creation Citation Report were used to extract data related to the year, authorship, country, type of document, institution, language, medium, and most cited documents on STEAM-EDU. To represent these data optimally in the manuscript, several inclusion criteria were established: year of publication (all literature between 2006 and 2020); language (x ≥ 2); area of knowledge (x ≥ 40); type of documents (x ≥ 32); institutions (x ≥ 21); authors (x ≥ 8); source of origin (x ≥ 17); countries (x ≥ 40); the four most cited documents.

**Fig. 2:**
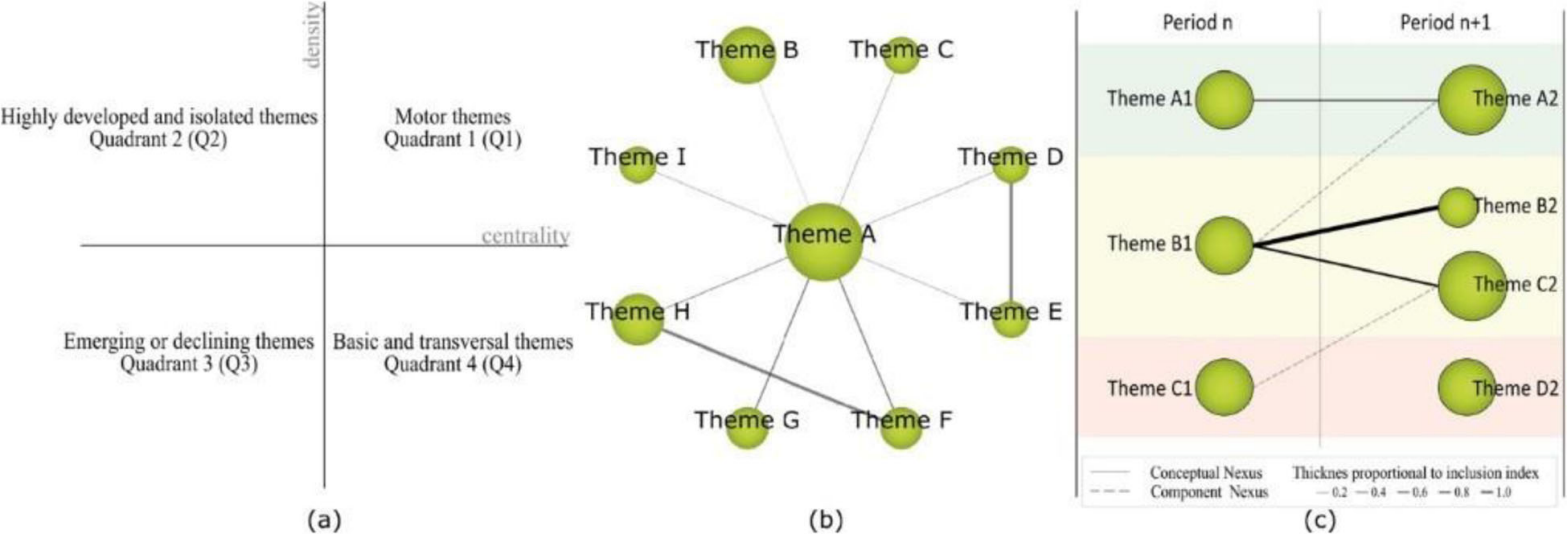
Structure and data of co-word analysis with SciMAT. *Note*. Strategic diagram (**a**). Thematic network (**b**). Thematic evolution (**c**)

Additionally, SciMAT was used to analyze the data concerning the dynamic and structural development, in a longitudinal manner, of the entire literature report. This consists of analyzing the evolution of a consolidated keyword in a topic over various time periods that have been previously defined (Herrera-Viedma et al. 2020). Similarly, SciMAT articulates the following processes:

- Recognition: The publication keywords (*n* = 3206) are studied. Co-occurrence maps are created by means of nodes. A network of co-words is generated. The most important keywords are established (*n* = 3070). The most significant terms and topics are clustered with a grouped algorithm.
- Reproduction: Strategic diagrams are designed to accommodate the terms according to their development in the literature. The diagrams are articulated in four quadrants (Q) (Fig. 2a): top right (Q1) = driving and relevant themes; top left (Q2) = entrenched or isolated themes; bottom left (Q3) = emerging or disappearing themes; and bottom right (Q4) = cross-cutting or underdeveloped themes. These diagrams are generated through the principles of density (internal strength) and centrality (connectivity between networks) (Montero-Díaz et al. 2018). Additionally, thematic networks (Fig. 2b) representing the main theme linked to other terms are elaborated.
- Determination: Time periods are established to classify the publications and to be able to analyze the development of nodes over time. The time periods are configured on the basis of the criterion of establishing a volume of publications with a certain similarity between them. In this study, three periods have been established (P_1_ = 2006–2015; P_2_ = 2016–2018; P_3_ = 2019–2020). However, for the study of authors, only one period has been established, i.e., from the beginning to the end (P_X_ = 2006–2020). To calculate the strength of associations between the periods, the number of common keywords or themes is used.
- Performance: The development of the themes is analyzed through the established time intervals (Fig. 2c). In addition, production indicators associated with inclusion criteria are delimited (Table 1).

**Table 1:** Production indicators and inclusion criteria

Configuration	Values
Analysis unit	Keywords: authors, keywords, Web of Science (WoS)
Frequency threshold	Keywords: P_1_ = (2), P_2_ = (2), P_3_ = (2)
	Authors: P_X_ = (4)
Network type	Co-occurrence
Co-occurrence union value threshold	Keywords: P_1_ = (2), P_2_ = (2), P_3_ = (2)
	Authors: P_X_ = (1)
Normalization measure	Equivalence index: eij = cij2/Root (ci − cj)
Clustering algorithm	Maximum size: 9; minimum size: 3
Evolutionary measure	Jaccard index
Overlapping measure	Inclusion rate

## Results

### Scientific output and production

**Fig. 3:**
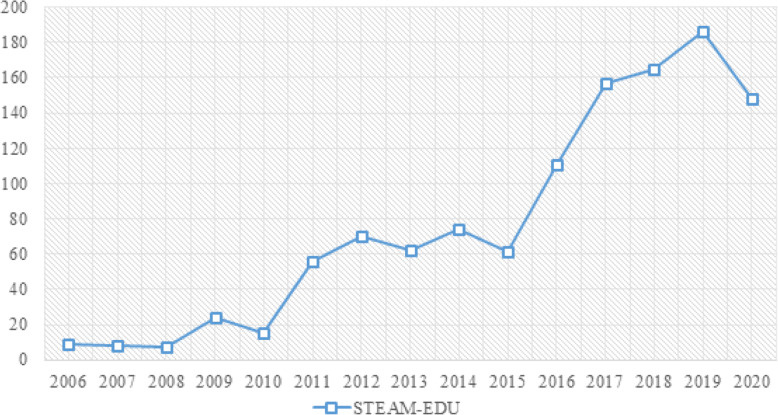
Evolution of scientific production

The scientific production collected on STEAM-EDU is from 2006, coinciding with its beginning in the scientific field, until 2020. Its evolution has been irregular, with three clearly differentiated stages. The first stage consists of the years 2006 to 2010, both included. During this period, scientific production did not exceed thirty manuscripts per year. The second stage is characterized by a substantial increase in scientific production. This stage covers the years 2011 to 2015 inclusive. In this period, around 70 scientific productions were generated per year. Production remained constant during these years. The third stage comprises the years 2016 to 2020, both inclusive. In this period, a continuous and increasing rise in scientific production was generated, reaching its peak in 2019. The year 2020 changed this trend, with a significant drop in production (Fig. 3).

English is undoubtedly considered the main language of STEAM-EDU scientific production. Other languages show a residual production compared to English (Table 2).

**Table 2:** Scientific languages used

Languages	n
English	1114
Spanish	20
Russian	5
Turkish	3

The STEAM-EDU studies mainly cover two areas of knowledge: Education Educational Research and Education Scientific Disciplines. Both areas focus mainly on education. The remaining knowledge areas present much smaller productions. It should be noted that the areas in which studies are collected focus their fields of research on engineering and computing (Table 3).

**Table 3:** Areas of knowledge

Denomination	n
Education Educational Research	794
Education Scientific Disciplines	480
Engineering Multidisciplinary	148
Computer Science Interdisciplinary Applications	63
Electrical and Electronic Engineering	45

The scientific community opts for research articles to present the most significant findings on STEAM-EDU. In addition, it is worth noticing the high volume of proceedings papers in this field of study (Table 4).

**Table 4:** Type of document

Denomination	n
Article	653
Proceedings paper	450
Book chapter	46
Review	33

In the STEAM-EDU studies, no single scientific institution can be identified as standing out above the rest in terms of the volume of output. The number of productions was even when we analyzed the institutions that occupy the top positions. The institution with the highest volume of production is the University System of Georgia (Table 5).

**Table 5:** Institutions

Denomination	n
University System of Georgia	30
State University System of Florida	27
Purdue University	24
University of North Carolina	24

Regarding authors’ individual output volume, the case is similar to that of the institutions. There is no one author who stands out above the rest. It is true that there are a few authors who show a slightly higher volume of production than the rest. This is the case of Bazler, J. and Vansickle, M., both with 14 productions (Table 6).

**Table 6:** Most prolific authors

Authors	n
Bazler, J.	14
Vansickle, M.	14
Freeman, J.	11
Herro, D.	9
Magerko, B.	9
McKlin, T.	9

In relation to the source of scientific production, it is noteworthy that the top positions are occupied by proceedings paper productions. The scientific journal that appears first in the ranking of scientific production is Computer Applications in Engineering Education, with 18 scientific productions on STEAM-EDU. This volume of production is much lower than that of ASEE Annual Conference Exhibition, which occupies the first place with a total of 55 scientific productions (Table 7).

**Table 7:** Source of origin

Source titles	n
ASEE Annual Conference Exposition	55
INTED Proceedings	41
EDULEARN Proceedings	38
ICERI Proceedings	38
Frontiers in Education Conference	32
Integrated STEM Education Conference	21
Computer Applications in Engineering Education	18

The USA is the country with the largest academic output on STEAM-EDU studies. Its volume of production is much higher than other countries (Table 8).

**Table 8:** Countries

Countries	n
USA	562
Spain	71
Australia	50
England	45

The citation volume of manuscripts that focus their studies on STEAM-EDU is low compared to other fields of study. In this case, the first manuscript to appear is the work of Connor et al. (2015), with a total of 39 citations, followed by the work of Sochacka et al. (2016), with 28 citations; the work of Quigley and Herro (2016), with 26 citations; and the work of Herro et al. (2017) with 8 citations (Table 9).

**Table 9:** Most cited articles

References	Citations
Connor, A.M.; Karmokar, S.; Whittington, C. (2015). From STEM to STEAM: Strategies for Enhancing Engineering and Technology Education. *International Journal of Engineering Pedagogy 5*, 37-47. Doi: 10.3991/ijep.v5i2.4458	39
Sochacka, N.W.; Guyotte, K.W.; Walther, J. (2016). Learning Together: A Collaborative Autoethnographic Exploration of STEAM (STEM plus the Arts) Education. *Journal of Science Education*, *105*, 15-42. Doi: 10.1002/jee.20112	28
Quigley, C.F.; Herro, D. (2016). “Finding the Joy in the Unknown”: Implementation of STEAM Teaching Practices in Middle School Science and Math Classrooms. *Journal of Science Education and Technology*, *25*, 410-426. Doi:10.1007/s10956-016-9602-z	26
Herro, D.; Quigley, C. (2017). Exploring teachers' perceptions of STEAM teaching through professional development: implications for teacher educators. *Professional Development in Education*, *43*, 416-438. Doi: 10.1080/19415257.2016.1205507	18

### Structural and thematic development

**Fig. 4:**
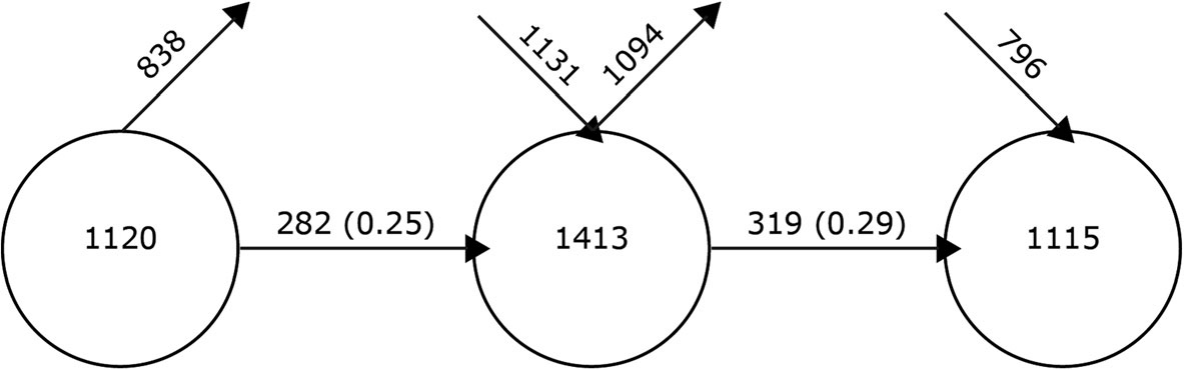
Keyword continuity between contiguous intervals

The development of keywords, shown in Fig. 4, shows, in the form of an X-ray, the development of keywords over the various periods. In this case, several data can be observed that are relevant for analysis. The first data is provided by the ascending arrows. These indicate the number of keywords that are not used in the following period. The second piece of information is shown by the descending arrows. These indicate the number of new keywords that are added in a given time period. The third piece of information is provided by the horizontal dates. These indicate the number of keywords overlapping between two periods. The last of the data is provided by the circles. These show the number of keywords used by the authors in that period. From these data, in this case, it can be seen that there is no established line of research over time. This is due to the percentage of overlap between continuous periods, reaching 25% in the case of 2006–2015 and 2016–2018 and 29% in the case of 2016–2018 and 2019–2020. In both cases, it is less than 30%. It is true that in the comparison of the last two time periods, the percentage increases. This may point to the establishment of a line of research in the not-too-distant future, but it is still in the process of formation.

The figures presented below show diverse and highly relevant information for this study. On the one hand, an interval diagram is presented, which determines the value of the themes obtained in the analysis of co-words in a given period. In this case, the analysis was based on Callon’s index (Callon et al. 1991), which generates a grouping of keywords and themes based on centrality (strength of the relationship between external links) and density (strength of the relationship between internal links). On the other hand, the analysis of academic performance is also presented. This data determines the value of various subjects according to their bibliometric indicators. These bibliometric indicators are the h-index, g-index, hg-index, and q2-index. The average number of citations per document is also examined in this study. Finally, a cluster network is presented. These clusters show the keywords or themes to which the various themes shown in the interval diagram are related.

**Fig. 5:**
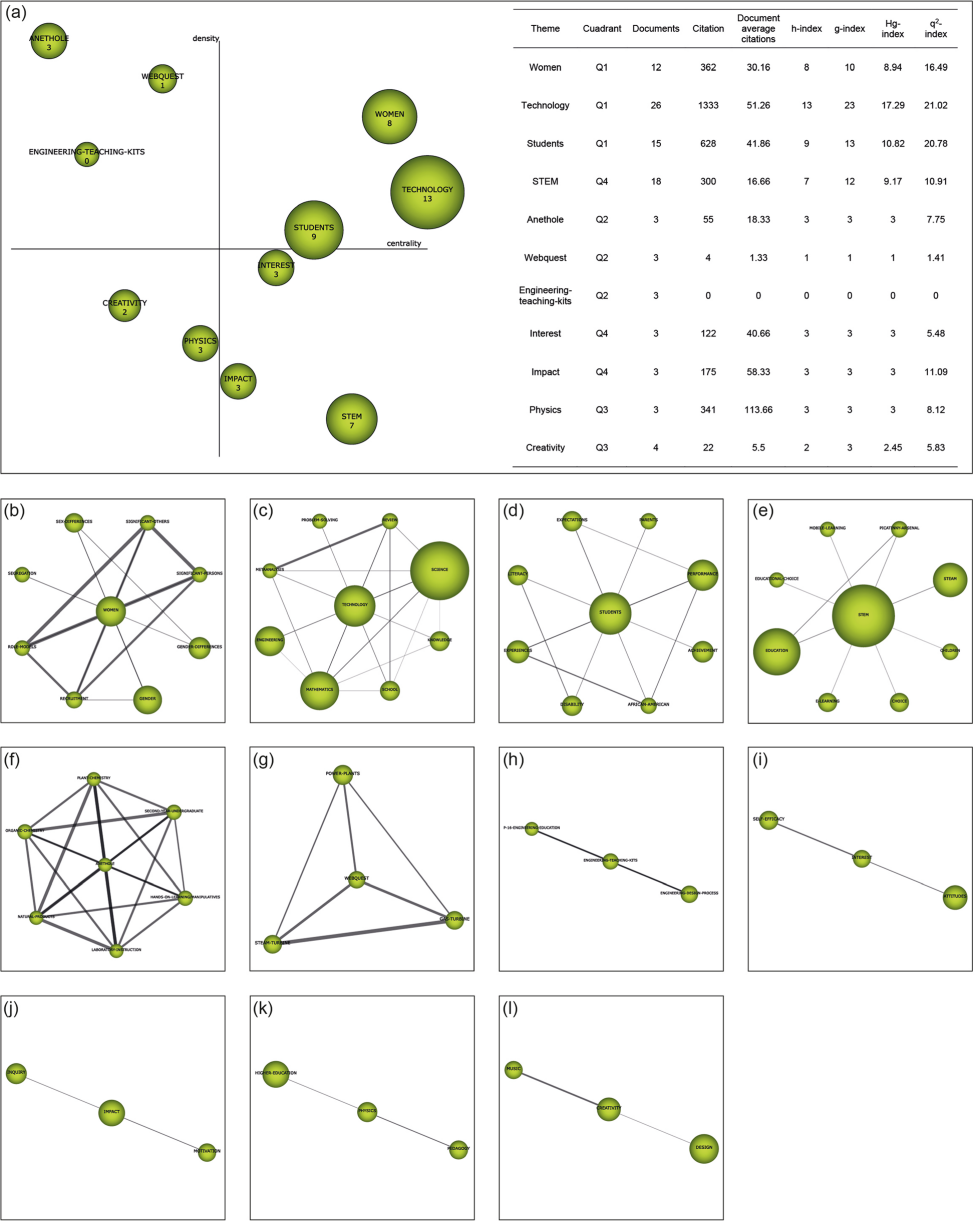
Strategic diagram of the 2006 to 2015 period. *Note*. **a** Strategic diagram (h-index) and performance from 2006 to 2015. Themes include **b** “women,” **c** “technology,” **d** “students,” **e** “STEM,” **f** “Anethole,” **g** “webquest,” **h** “engineering-teaching-kits,” **i** “interest,” **j** “impact,” **k** “physics,” and **l** “interest”

Beginning the analysis with the first time period established (2006–2015), it can be seen that “technology” is the subject with the highest bibliometric value, being much higher than those of the rest of the subjects. Furthermore, it coincides with the fact that this subject, “technology,” is located as a driving theme in this period. In addition, there are two other subjects considered to be driving forces in this period, namely “women” and “students.” If we analyze the cluster networks of these driving themes, we can see that “women” is related to “sex-differences,” “significant-others,” “significant-persons,” “gender-difference,” “gender,” “recruitment,” “role-models,” and “segregation”; “technology” relates to “problem-solving,” “review,” “science,” “knowledge,” “school,” “mathematics,” “engineering,” and “meta-analysis”; and “students” relates to “expectation,” “parents,” “performance,” “achievement,” “African-American,” “disability,” “experiences,” and “literacy.” This period focuses mainly on women and their relationship to STEAM, specifically on possible gender differences in technology and its influence on student development. In addition, there is a focus on the involvement of STEAM in people of diverse races and diverse abilities (Fig. 5).

**Fig. 6:**
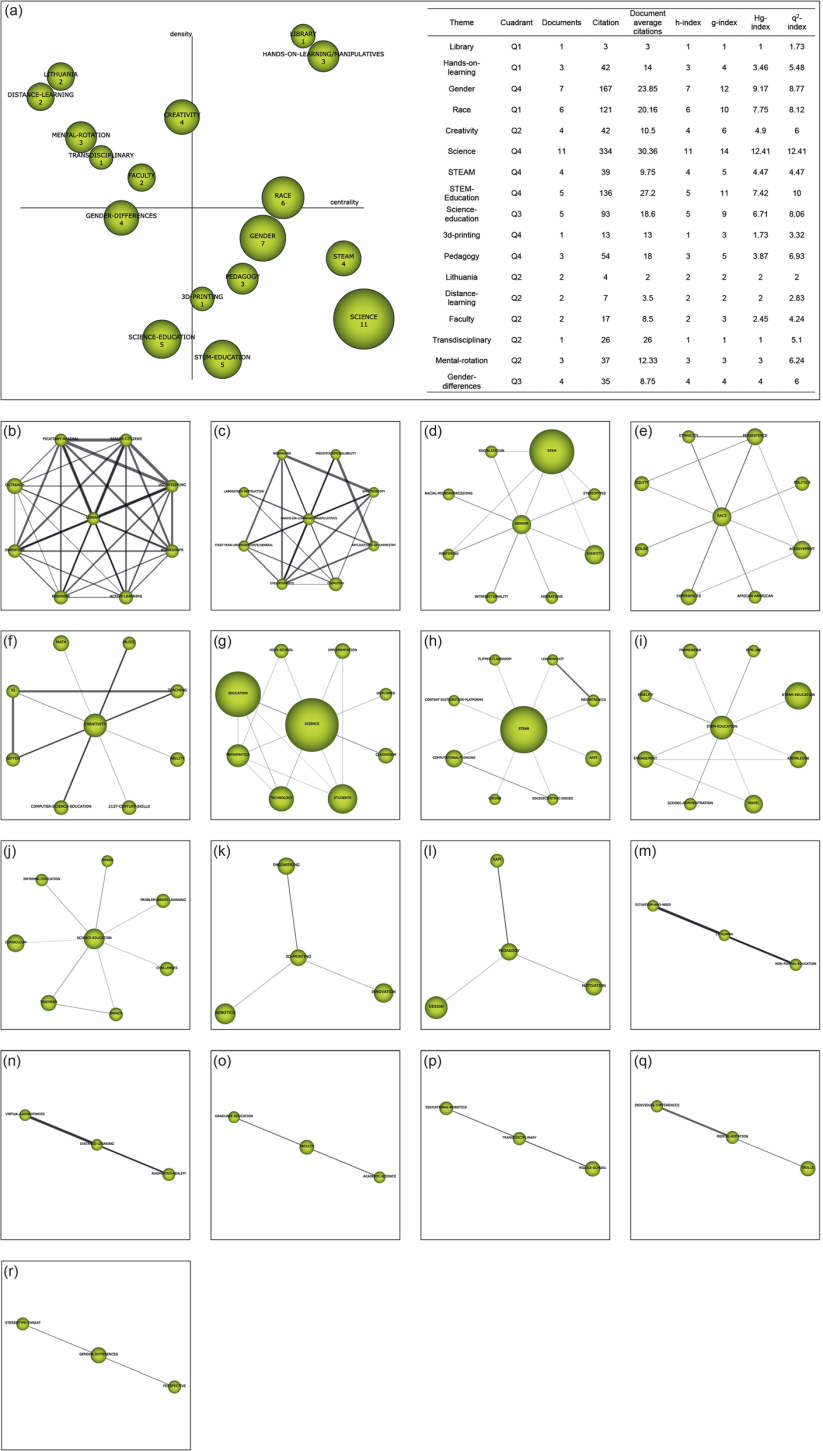
Strategic diagram of the 2016 to 2018 period. *Note*. **a** Strategic diagram (h-index) and performance from 2016 to 2018. Themes include **b** “library,” **c** “hands-on-learning/manipulatives,” **d** “gender,” **e** “race,” **f** “creativity,” **g** “science,” **h** “STEAM,” **i** “STEM-Education,” **j** “Science-education,” **k** “3d-printing,” **l** “pedagogy,” **m** “Lithuania,” **n** “distance-learning,” **o** “faculty,” **p** “transdisciplinary,” **q** “mental-rotation,” and **r** “gender-differences”

In the second period analyzed (2016–2018), the subject with the highest bibliometric value is “science,” well above the rest. This subject has the peculiarity of not being considered a driving theme in this period, although, given its bibliometric values and its importance in this period, it should be taken into account. The topics considered as driving themes in this period are “library,” which is related to “picatinny-arsenal”, “senior-citizens,” “volunteering,” “workshops,” “active-learning,” “engineer,” “invention,” and “outreach”; “hands-on-learning/manipulatives,” which relates to “nonmajors,” “precipitation/solubility,” “spectroscopy,” “applications-of-chemist,” “chemistry,” “dyes/pigments,” “first-year-undergraduate/general,” and “laboratory-instruction”; and “race,” which relates to “ethnicity,” “persistence,” “politics,” “achievement,” “African-American,” “experiences,” “colour,” and “equity.” This period is characterized by a research focus on the use of STEAM in education outside the formal setting, focusing studies on retired people, volunteers, and other non-formal education settings. It also focuses research on the influence of STEAM on people of different races, as well as identifying equity in access to STEAM. In this period, moreover, chemistry studies emerged as the branch of education that began to take STEAM most strongly into use (Fig. 6).

**Fig. 7:**
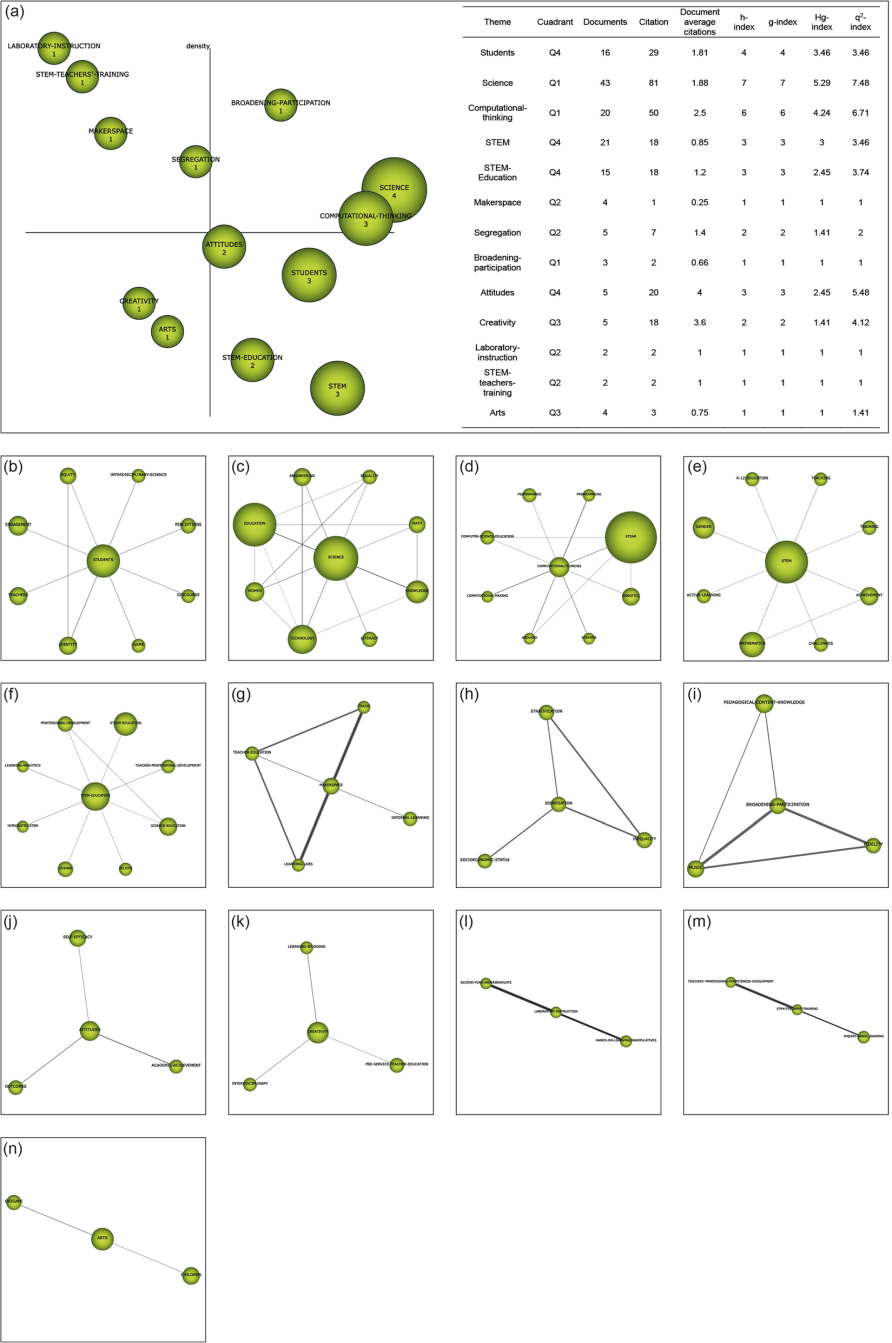
Strategic diagram of the 2019 to 2020 period. Note: **a** Strategic diagram (h-index) and performance from 2019 to 2020. Themes include **b** “students,” **c** “science,” **d** “computational-thinking,” **e** “STEM,” **f** “STEM-Education,” **g** “makerspace,” **h** “segregation,” **i** “broadening-participation,” **j** “attitudes,” **k** “creativity,” **l** “laboratory-instruction,” **m** “STEM-Teachers-training,” and **n** “arts”

In the third and final period (2019–2020), two subjects analyzed stand out from the rest in terms of academic performance, namely “science” and “computational-thinking.” Moreover, both themes are considered as driving themes in this period, together with “broadening-participation.” Analyzing the cluster’s network, we observe that “broadening-participation” is related to “pedagogical-content-knowledge,” “music,” and “fidelity”; “science” is related to “engineering,” “equality,” “math,” “knowledge,” “literacy,” “technology,” “women,” and “education”; and “computational-thinking” relates to “performance,” “programming,” “STEAM,” “robotics,” “systems,” “arduino,” “computational-making,” and “computer-science-education”. In this period, the focus of the scientific community is on the involvement of students in the teaching and learning processes developed with STEAM, on the pedagogical knowledge of teachers when using STEAM, on the influence of STEAM in the field of science and on computational thinking. Furthermore, in this last period, the themes “creativity” and “arts” should be kept in mind, since their location in the diagram places them as unknown themes. This is because they may disappear from the STEAM-EDU research line or be the next driving themes in this field of study (Fig. 7).

The themes resulting from the co-word analysis and their location in the strategy diagram, represented in Figs. 5, 6, and 7, are shown in Table 10. This table shows the position of the themes in the strategic diagram with their respective centrality and density indices. In other words, this table provides an overview of all of the themes appearing in the three time diagrams established in this research. With this table, it is possible to observe whether there is continuity of the same themes in the three time periods and the relevance of the themes in each of the time periods. It also shows the changes in the themes from one time period to another. As can be seen, there is no conceptual gap in this field of study. This is due to the fact that the theme “creativity” is repeated in all the established time periods. This places the theme “creativity” as the common thread of the research on STEAM-EDU, although its location in the various time diagrams does not denote it as a driving theme at any time.

**Table 10:** Principal research themes related to science, technology, engineering, art, and mathematics in education (STEAM-EDU) from 2006 to 2020

	P1 (2006–2015)	P2 (2016–2018)	P3 (2019–2020)
Women	Q1(34.27/71.98)		
Technology	Q1(45.45/26.24)		
Students	Q1(23.62/17.14)		Q4(30.74/7.24)
STEM	Q4(33.86/6.13)		Q4(34.15/3.49)
Anethole	Q2(0/226.98)		
WebQuest	Q2(4.05/87.5)		
Engineering teaching kits	Q2(0.93/45)		
Interest	Q4(20.12/16.67)		
Impact	Q4(10.17/7.9)		
Physics	Q3(5.57/9.33)		
Creativity	Q3(2.35/16.2)	Q2(10.99/32.88)	Q3(2.34/6.89)
Library		Q1(39.64/213/01)	
Hands-on learning		Q1(45.92/128.9)	
Gender		Q4(26.69/11.08)	
Race		Q1(32.76/16.69)	
Science		Q4((72.23/7.38)	Q1(39.91/12.57)
STEAM		Q4(60.75/10.22)	
STEM Education		Q4(18.96/5.91)	Q4(19.39/4.51)
Science Education		Q3(9.98/6.68)	
3D printing		Q4(17.46/8.41)	
Pedagogy		Q4(19.15/10.14)	
Lithuania		Q2(0.92/77.78)	
Distance learning		Q2(0.7/66.67)	
Faculty		Q2(2.81/19.05)	
Transdisciplinary		Q2(1.73/19.38)	
Mental rotation		Q2(1.71/28.57)	
Gender differences		Q3(2.24/11.11)	
Computational thinking			Q1(35.4/10.03)
Makerspace			Q2(1.84/55)
Segregation			Q2(5.92/37.08)
Broadening participation			Q1(21.74/57.64)
Attitudes			Q4(10.76/10)
Laboratory instruction			Q2(0/77.78)
STEM Teachers training			Q2(0/60)
Arts			Q3(5.36/5.13)

*Note*: (X/Y), *X*, centrality; *Y*, density

**Fig. 8:**
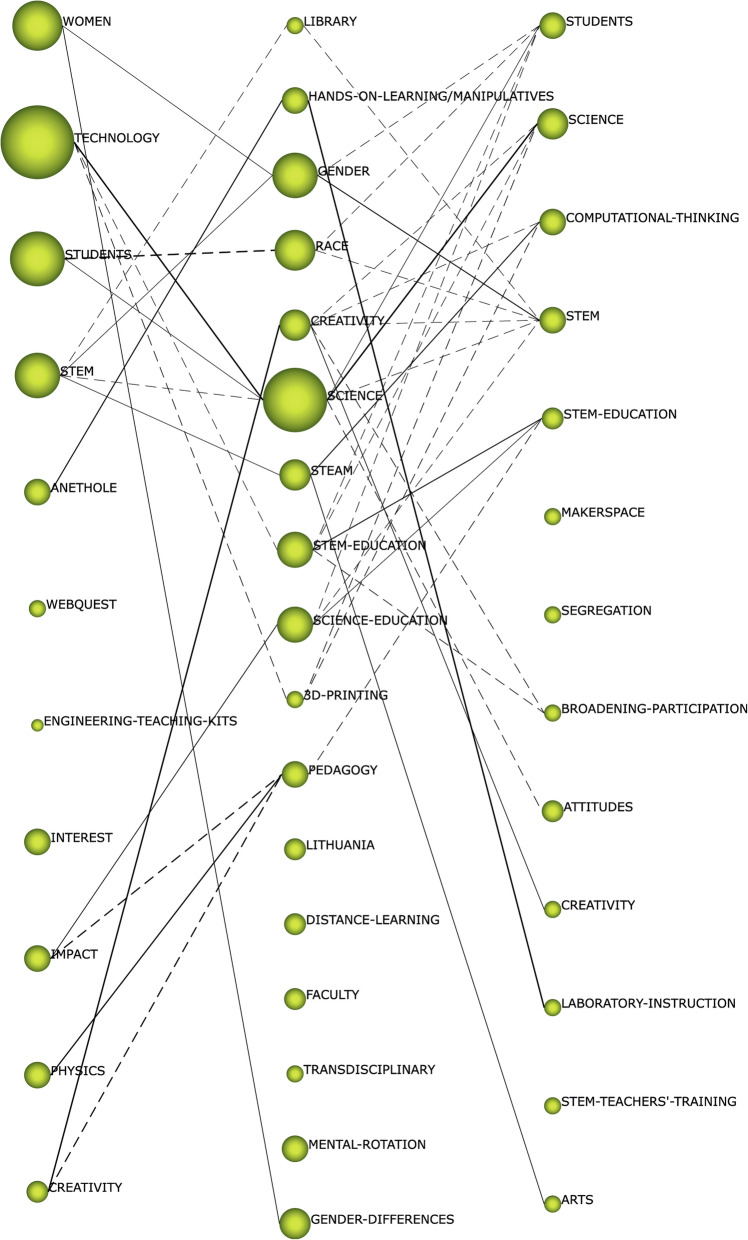
Thematic evolution by h-index

The thematic evolution of STEAM-EDU-related research was generated using the Jaccard index (Real and Vargas 1996). This indicator generates relationships between themes, taking into account whether the relationship established between themes is based on keywords or on other themes. If the connection is thematic, the connection is represented in the graph by a continuous line. In this case, it is considered a conceptual connection. If the connection is based on keywords, the link is represented in the graph by a broken line. In this case, it is considered a non-conceptual connection. In addition, the thickness of the connecting line must be taken into account. The thickness indicates the number of overlapping keywords or themes. In this case, the greater the thickness is, the greater the number of coincidences is between contiguous themes. If we analyze Fig. 8, we can observe several aspects that need to be highlighted. Firstly, the number of connections between contiguous themes is low, indicating a lack of connections between the various lines of research established in STEAM-EDU studies. Secondly, more non-conceptual than conceptual connections are observed, which reinforces the above. This is another indicator of the lack of relationships between subjects. Thirdly and finally, there are three clearly defined lines of research. One of these is “creativity,” which is represented in all three time periods. This line of research, although constant over time, is not the strongest in this field of study. The other two lines of research do strongly represent the STEAM-EDU line of research, namely “anethole_hands-on-learning_laboratory-instruction” and “technology-science-science,” which focus their studies on the scientific field and on training in the branch of science.

### Authors with the highest relevance index

**Fig. 9:**
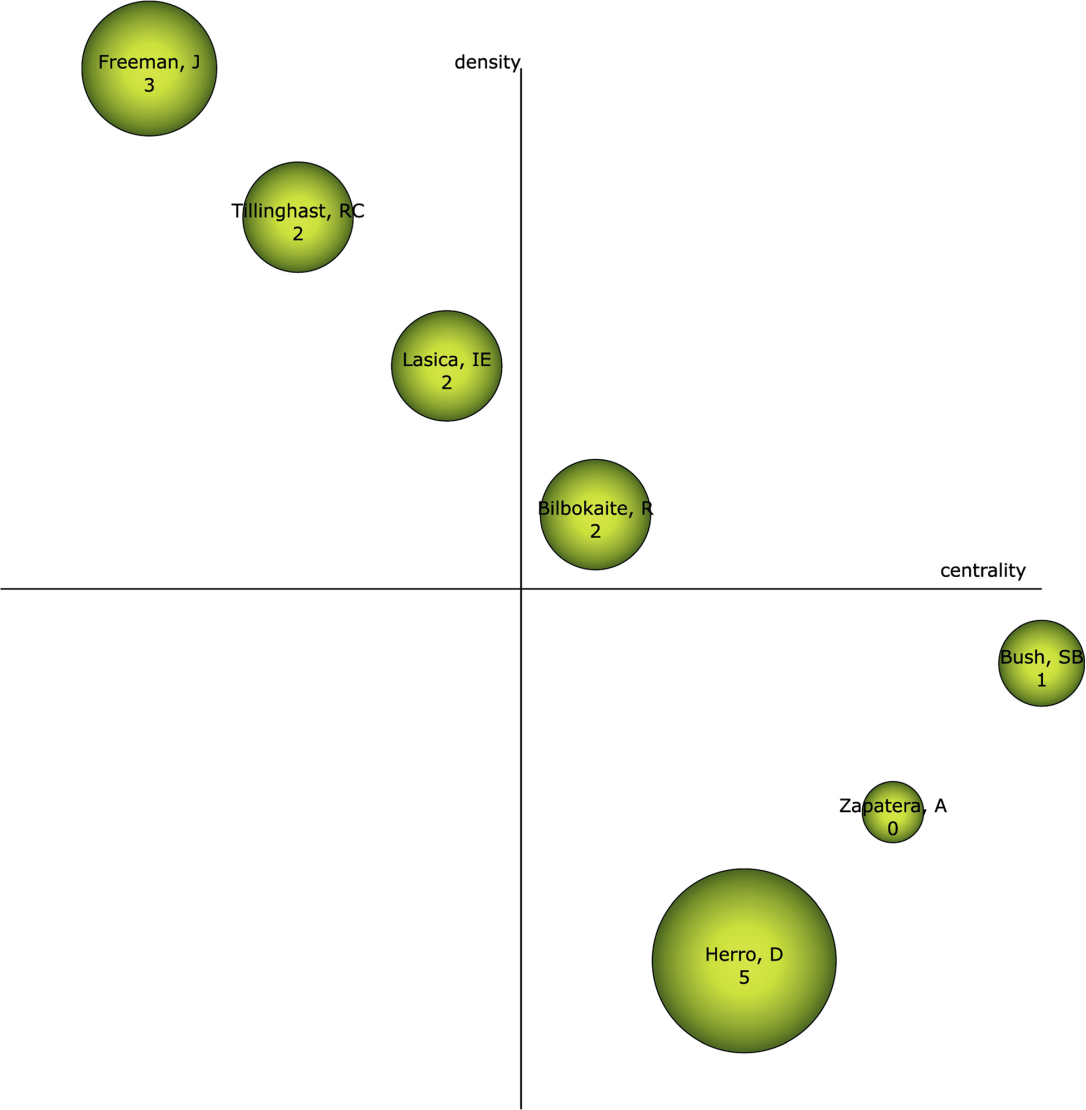
Strategic diagram by h-index of authors of all scientific output

Taking into account the position of the authors in the diagram represented in Fig. 9, it can be established that Bilbokaite, R. is the leading author in this field of study and should be considered relevance and interest in the scientific community. Herro, D. should also be taken into account as she has the highest bibliometric value of all the authors resulting from the analysis. It is noteworthy that in the field of study on STEAM-EDU, there is no author in particular who could be relevant or stand out in the coming years. In other words, the resulting data, to date, indicate that in the coming years, there are no authors in particular who will stand out more than others in this field of study.

## Discussion

Digital media and tools are increasingly entering today’s society, impacting the global economy and labor market. There is an increasing demand for technology-related professions, as machines are taking over the work of humans (Anisimova et al. 2020; Anito and Morales 2019; Casado and Checa 2020). These needs call for a redefinition of the curriculum at all stages of education, where technologies are also being integrated into the classroom, in order to train citizens of the future. STEAM projects (Angel and Salgado 2018; Bush et al. 2020; Chu et al. 2018; Colucci-Gray et al. 2019; Conradty and Bogner 2020; Dolgopolovas and Dagiene 2021; Greca et al. 2021; Herro et al. 2017; Lin and Tsai 2021; Mengmeng et al. 2019; Perignat and Katz-Buonincontro 2018; Togou et al. 2019; Webb and LoFaro 2020) were born over the last decade from the union of STEM subjects and the arts.

Emerging methodologies that apply STEAM in education have emerged in recent years as a pedagogical alternative and provide a more holistic and engaging education (Casado and Checa 2020; Chen and Huang 2020; Chien and Chu 2017; Conradty et al. 2020; De la Garza 2019; Harris and Bruin 2017; How and Hung 2019; Jesionkowska et al. 2020; Juskeviciene et al. 2020; Kim 2016; López-Belmonte et al. 2021; Lytle et al. 2019; Moreno-Guerrero et al. 2021a, 2021b; Rodríguez et al. 2020; Segarra et al. 2018; Taljaard 2016; Tan et al. 2020; Wu et al. 2021). It develops critical and creative thinking, produces motivation in students, and reduces the gender gap due to its transdisciplinary, interdisciplinary, and multidisciplinary teaching process. Research on STEAM curricula (Bassachs et al. 2020; Diego-Mantecón et al. 2020; Hadinugrahaningsih et al. 2017; Hong et al. 2020; Knochel 2019; Ng and Fergusson 2020; Oliveros-Ruiz 2019) can offer an educational pathway for different teaching methodologies and successfully demonstrate that art and creativity promote motivation (Lu and Ma 2019).

Studies on STEAM-EDU have been generated from 2006 to the present day, uninterruptedly, although the interest generated in the scientific community has been irregular. In this case, three clearly distinct stages can be observed: a first stage of low production, between 2006 and 2010, inclusive; a second stage of average production, from 2011 to 2015, inclusive; and a third stage, with production rising steadily until 2019. In 2020, production levels reduced, dropping to 2017 levels. This has been observed in other fields of study, such as robotics (López-Belmonte et al. 2021) or augmented reality (López-Belmonte et al. 2020a, 2020b). This coincides with the year of the COVID-19 pandemic. This may be the main reason why there has been such a reduction in production in various fields of study, especially in the field of education.

The performance of the scientific production on STEAM-EDU shows that English is the language mainly used by the scientific community. This is mainly due to the WoS database, which collects studies from in the English language. Furthermore, it should be noted that English is the second language in many countries. This means that scientific studies written in English reach more people and a wider spectrum of the scientific community.

The main area in which studies on STEAM-EDU are collected is Education Educational Research. That is, the majority of studies are compiled in the purely educational field, although other branches of knowledge, such as engineering and computer science, have also established lines of research on STEAM-EDU. This is due to the fact that both branches of knowledge make use of STEAM for the development of their curricular contents.

The type of document used by scientists to present their research is research articles, although proceedings papers are also widely used. When analyzing the main sources of origin, we can see that proceedings papers are the most popular, such as the ASEE Annual Conference Exposition or INTED Proceedings. This can be interpreted in several ways. On the one hand, it is considered that the line of research is established over time, since research articles show maturity in the fields of study. On the other hand, however, proceedings papers offer trends or new perspectives on changes in the field of study. In this case, it can be said that in STEAM-EDU, there is an established research trend, but new lines of research are being generated.

The main institutions conducting research on STEAM-EDU are located in the USA. Moreover, the USA is the main producer of manuscripts in this line of research. Among the institutions with the highest volume of production are the University System of Georgia and the State University System of Florida.

With respect to authors, there are two who stand out for having the highest volume of production in STEAM-EDU studies, namely Bazler, J. and Vansickle, M., with 14 manuscripts each. On the other hand, Bilbokaite, R. is the author considered the driving force in this field of study, although it is Herro, D. who presents the highest bibliometric value. Nevertheless, any of these authors could be a reference in the field of study on STEAM-EDU.

Among the most cited articles in the STEAM-EDU research line is that of Connor et al. (2015), which is oriented along the lines of engineering and educational technology. This manuscript has 39 citations to date. As can be seen, the volume of citations is relatively low in this field of study compared to that for other research in the field of education (Carmona-Serrano et al. 2021; López-Belmonte et al. 2020a, 2020b).

Continuing with the analysis, the evolution of keywords showed that the level of coincidence between time periods is relatively low, given that the percentage of coincidence is less than 30%. Taking this into account, it can be considered that there is no established line of research over time. It is true that in recent years, the level of coincidence has increased, but it is insufficient. These data confirm what was indicated above in relation to proceedings papers, where new trends are being generated and new lines of research are being established.

In relation to the established time periods, various trends can be observed in the field of STEAM-EDU studies. In the first period (2006–2015), the topic of “technology” stands out as a reference among the scientific community. In addition, the focus of this period is on the gender of students and the influence of the use of technological resources in students’ educational processes. Another noteworthy aspect in this period is the studies on the influence of STEAM on people according to their race and abilities. In the second period (2016–2018), research focuses on both formal and non-formal education. In addition, the influence of STEAM on people according to their race continues to be analyzed. It is also worth noting that in this period, chemistry studies emerged as the main field exploiting this pedagogical strategy. In the third and final period (2019–2020), STEAM-EDU studies focus on teachers’ pedagogical knowledge—that is, on their pedagogical skills and knowledge to be able to implement STEAM in teaching and learning processes. In this context, the current teaching methodologies used by teachers in this digital age, due to training deficiencies, do not reflect the learning that students will face in the future (Max et al. 2020), which is critical to a country’s economy, and there are concerns about how they are being delivered, focusing on science and side-lining societal and environmental concerns (Colucci-Gray et al. 2017). In addition, computational thinking is gaining momentum in this field of study. STEAM-EDU research also focuses on the field of science. Taking into account the results achieved in the co-word analysis, it can be established that the topics “creativity” and “arts” will, with relative probability, be the new trends in the STEAM-EDU field of study. The terms art and creativity create links between the mind and nature of individuals. They enable reflection on a constantly changing world (Colucci-Gray 2019) and increase the sense of perception and social competence as a method of cooperation through individual responsibility and interaction, in addition, stimulate makerspaces and multidisciplinary work teams, in order to obtain a more creative society (Bassachs et al. 2020). Through STEAM-EDU, students can ask questions about their position in the world (Biesta 2015).

In the thematic evolution, taking into account the established time periods, it can be seen that there is no conceptual gap in this STEAM-EDU line of research. This is because the theme of “creativity” is repeated in all three periods. In principle, it can be indicated that the common thread of STEAM-EDU research is “creativity,” but there are other lines of research that are more relevant and stronger in STEAM-EDU studies, such as “anethole_hands-on-learning_laboratory-instruction” and “technology-science-science.” In other words, the focus of research, taking into account the periods as a whole, is the science–technology branch. Another aspect to note is that the number of connections between contiguous themes is low, indicating a lack of connections between the various lines of research established in STEAM-EDU studies. In addition, there are more non-conceptual than conceptual connections, which reinforces the above. Both aspects confirm the lack of connections between the various lines of research.

Arts education as a complement to STEM can be beneficial (Bazler and Van Sickle 2017), albeit complex. With this article, we contribute to the recommendations of Colucci-Gray et al. (2019) on STEAM, opening “new lines of research and arguments on STEAM literature to teach new content, expand the means to understand and cultivate scientific and artistic creativities, and make explicit key connections to the materiality of practices” (p. 1), with this article being the first step in the research related to pedagogical practices in the different disciplines covered by the term, through the arts, to concretize its effectiveness.

## Conclusions

It can be concluded that STEAM-EDU studies do not have an established and robust line of research over time, although it can be observed that the trends in this aspect are focused on the scientific branch of education. In addition, the topics of study on STEAM-EDU include aspects related to gender differences, the influence of STEAM-EDU on people of different races, the skills developed by students, and teacher training to implement teaching and learning processes with STEAM-EDU; above all, in recent times, the STEAM-EDU line of research is being related to computational thinking.

The limitations of this study are directly related to the sorting of documents from the WoS database. The term STEAM appears in this database as an acronym for various concepts, which has made it difficult to study and analyze it. This required extra effort on the part of the research team to filter those documents that coincided with the construct that we wanted to analyze and that had been established in the objectives of the manuscript. Moreover, it is important to note that the time periods examined since 2006 show large differences depending on the interval analyzed. On the other hand, it should be added that the dimensions of this research have been established by the researchers themselves on the basis of their own criteria in order to offer coherent results in accordance with the WoS. This fact allows the results obtained to be extrapolated to other research, but with caution, since, just as other research may be enriched by the contributions of this manuscript, a subsequent study may counteract the dimensions and modify the connections between the themes analyzed. In other words, the results offered in this study are a current snapshot of the term STEAM in the WoS database that may be modified over time due to the dynamism of this database. As for the future lines of work offered by this study, it would be interesting to know the level of association of the term STEAM with other areas of knowledge and its implications with the gender variable, as well as to analyze, in depth, the relationship between computational thinking and STEAM.

## Study implications

According to the previous literature review, this study is the first work to analyze the term STEAM from a deeper perspective through scientific mapping of the concept. This has provided a series of results that give rise to various implications of both a theoretical and practical nature. From a theoretical point of view, the study contributes to increasing the scientific literature on STEAM in the educational field. The results also make it possible to determine the trends that are developing in this line of research. At the same time, it shows the scientific community the main lines of work that researchers have developed since their appearance in the WoS database. In addition, the different bibliometric indicators have revealed the emerging topics that are related to the STEAM construct. This diversity of information can serve as a guide and model for future studies that require data related to STEAM and variables such as authors, institutions, medium and areas of publication, languages, most cited documents, or other more particular information offered by this study. Another relevant aspect in the theoretical field for future lines of research is that studies should focus on the development of creativity, as has been performed up to now, and more strongly in studies related to the scientific field and training in the field of science.

In the same way, this study shows a series of practical implications related to the term STEAM for the educational field, which directly affect groups such as teachers, students, researchers, training institutions, or the technological tools themselves for training in the disciplines covered by the acronym. Thus, the studies and research that are currently being developed are aimed at the development of computational thinking; at broadening student participation, taking the inclusion of all without distinction of gender as a starting point; at training teachers for effective and interdisciplinary teaching of the different areas that make up the STEAM acronym; at the development of positive attitudes towards the development of computational thinking; at the development of positive attitudes towards the study of these areas and their integration as an economic and knowledge engine; at the creation of work spaces that allow for the development of each student's potential; and, finally, at the development of creativity as the backbone for the interconnection of knowledge and the resolution of problems in a globalized and interdisciplinary manner. All of this can serve as a guide for different institutions to promote training actions that improve the teaching and learning processes of the different disciplines. Furthermore, it can favor educational practices that provide students with holistic knowledge and the ability to solve problems by combining the knowledge provided by the different areas of study.

## Data Availability

The datasets used and/or analyzed during the current study are available from the corresponding author on reasonable request. Data are contained within the article.

## Abbreviations

AI: Artificial intelligence
ASEE: American Society for Engineering Education
COVID-19: *Coronavirus disease*
CT: Computational thinking
INTED: International Technology, Education and Development Conference
JCR: Journal Citation Reports
SciMAT: Science Mapping Analysis Tool
STEAM: Science, Technology, Engineering, Art and Math
STEAM-EDU: Science, Technology, Engineering, Art and Math in education
STEM: Science, Technology, Engineering, and Math
WoS: Web of Science
PRISMA: Preferred Reporting Items for Systematic reviews and Meta-analyses
